# Metabolomic signature reveals dysregulated lipoprotein profile in m.3243A>G carriers: a case-control study

**DOI:** 10.1007/s11306-026-02503-8

**Published:** 2026-07-03

**Authors:** Simone Rask Nielsen, Hien Thi Thu Nguyen, Malene Pontoppidan Stoico, Christina Brock, Kurt Højlund, Inge Søkilde Pedersen, Anja Lisbeth Frederiksen

**Affiliations:** 1 Department of Clinical Genetics, Aalborg University Hospital, Aalborg, Denmark; 2 Department of Clinical Medicine, Aalborg University, Aalborg, Denmark; 3 Department of Molecular Diagnostics, Aalborg University Hospital, Aalborg, Denmark; 4 Mech-Sense, Department of Gastroenterology and Hepatology, Aalborg University Hospital, Aalborg, Denmark; 5 Steno Diabetes Center Odense, Odense University Hospital, Odense, Denmark; 6 Department of Clinical Research, University of Southern Denmark, Odense, Denmark; 7 Department of Clinical Genetics, Odense University Hospital, Odense, Denmark

**Keywords:** Mitochondria, m.3243A>G, Mitochondrial inherited diabetes and deafness, Diabetes mellitus, Metabolomics, Lipoproteins

## Abstract

**Introduction:**

The pathogenic mitochondrial gene variant m.3243A>G disrupts oxidative phosphorylation and is associated with insulin resistance, both of which may be linked to unfavorable lipid metabolism. However, the metabolic alterations in m.3243A>G carriers, including what differentiates those with and without diabetes, remain incompletely understood.

**Objectives:**

To investigate metabolomic profiles in fasting serum and urine samples from m.3243A>G carriers compared to healthy controls.

**Methods:**

Metabolomic profiling of serum and urine samples using nuclear magnetic resonance-based metabolomics in m.3243A>G carriers (*n* = 28) was compared to healthy controls matched for age and sex. Additionally, profiles from m.3243A>G carriers with diabetes (*n* = 16) were compared with carriers without diabetes (*n* = 12) to identify potential metabolites associated with the presence of diabetes.

**Results:**

Twenty-five metabolites in serum and 16 in urine were identified as metabolites separating m.3243A>G carriers from healthy controls. The m.3243A>G carriers presented with increased triglycerides across lipoprotein particles and altered very-low-density lipoprotein concentrations and composition. In addition, there were alterations in metabolites from a number of metabolic pathways, including glycolysis, the tricarboxylic acid cycle, glutathione, one-carbon, and nucleotide metabolism. A three metabolite-urine signature (uracil, hypoxanthine, and 1-methylnicotinamide) demonstrated discriminating potential between m.3243A>G carriers and controls in exploratory machine learning analyses (area under the curve values 0.94–0.99 and cross-validation prediction of 0.81–0.93). Among m.3243A>G carriers, branched-chain amino acids were higher in individuals with diabetes compared with carriers without diabetes.

**Conclusion:**

Dysregulated lipoprotein metabolism represents a significant metabolic fingerprint of m.3243A>G carriers. Furthermore, higher levels of branched-chain amino acids may be associated with the presence of diabetes.

**Supplementary Information:**

The online version contains supplementary material available at https://doi.org/10.1007/s11306-026-02503-8.

## Introduction

The clinical phenotype associated with the m.3243A>G mitochondrial DNA variant is highly heterogeneous, complicating both diagnostics and clinical management. Located in the *MT-TL1* gene, the m.3243A>G variant [MIM:590050] results in impaired mitochondrial oxidative phosphorylation (OXPHOS) with reduced adenosine triphosphate production and secondary effects on multiple metabolic pathways (Esterhuizen et al., 2017). Notably, each mitochondrion contains multiple copies of the mitochondrial genome. The proportion of m.3243A>G relative to wild-type variants, termed heteroplasmy, is positively associated with more severe phenotypes (Wallace, 2018). Despite increasing recognition of the clinical manifestations of m.3243A>G-associated disease, the systemic metabolic alterations underlying its pathophysiology remain incompletely understood. These gaps can be addressed using metabolomics, which is a comprehensive analysis and quantification of small-molecule metabolites in biological samples. Metabolomics reflects the downstream endpoints of cellular processes and provides a powerful approach for identifying disease-related metabolic shifts. When combined, blood and urine metabolomics expand the metabolic profile, enabling a more comprehensive assessment of disease-related metabolic alterations. Among circulating metabolic factors are lipoproteins, including very low-density lipoprotein (VLDL)s, which transport triglycerides, cholesterol, and other lipids between organs, thereby facilitating the distribution of fatty acids (Borén et al., 2022). These fatty acids undergo mitochondrial β-oxidation, producing acetyl-CoA for the citric acid cycle, and NADH and FADH₂ that fuel OXPHOS. Consequently, m.3243A>G-associated impairment of OXPHOS may influence lipid metabolism.

Dysregulation of lipoproteins is associated with an increased risk of developing type 2 diabetes, a metabolic disorder with insulin resistance (Ahola-Olli et al., 2019; Bragg et al., 2022; Seah et al., 2022). Importantly, m.3243A>G carriers have a high risk of developing diabetes mellitus, commonly referred to as maternally inherited diabetes and deafness (MIDD), which is characterized by insulin resistance combined with impaired insulin secretion and absence of overweight (El-Hattab et al., 2014; Langdahl et al., 2019; Murphy et al., 2008). Previous metabolomics studies in m.3243A>G carriers *with* diabetes have reported pronounced dysregulations in energy metabolites, including elevated circulating triglycerides and alterations in several metabolites involved in glucose metabolism and fatty acid oxidation in serum and urine (Cao et al., 2025; Esterhuizen et al., 2021). In contrast, m.3243A>G carriers *without* diabetes appear to exhibit less pronounced metabolic disturbances, including triglyceride levels comparable to those of healthy individuals (Esterhuizen et al., 2021; Langdahl et al., 2019). However, it remains unclear whether m.3243A>G carriers with and without diabetes have an altered metabolic profile, including lipoprotein subclasses, their lipid concentrations, and composition.

In this study, we aimed to investigate the metabolomic profile of m.3243A>G carriers with a particular focus on lipoprotein metabolism and compared them with age- and sex-matched healthy controls using a high-throughput quantitative nuclear magnetic resonance (NMR) metabolomics platform in serum and urine samples.

## Materials and methods

### The design and study cohort

This cross-sectional case-control study included adult carriers of the m.3243A>G variant, recruited from the Department of Clinical Genetics at Aalborg University Hospital, and from an established Danish cohort of m.3243A>G carriers (Langdahl et al., 2018).

Healthy control participants were recruited through the Blood Donation Centre at Aalborg University Hospital and via public advertisement on social media platforms. Healthy controls were defined as individuals without known acute or chronic disease and not receiving medication known to influence metabolic function. This was screened through self-reports and the electronic medical journal. Controls were pairwise matched to m.3243A>G carriers by age and sex and were required to have a body mass index below 26 kg/m² to minimize potential confounding effects of obesity-related metabolic disturbances.

### Clinical assessment

All participants underwent a clinical assessment, including measurements of height, weight, and current medication use. For m.3243A>G carriers, phenotypic manifestations associated with mitochondrial disease were assessed through structured interviews and a review of electronic medical records. Clinical manifestations evaluated included: diabetes mellitus (defined according to the criteria established by the American Diabetes Association (ADA) (2025)), ataxia, epilepsy, stroke-like episodes, peripheral neuropathy, myopathy, hearing impairment, cardiomyopathy, hypertension, and nephropathy. These assessments were used to characterize the clinical phenotype of the m.3243A>G carrier cohort.

### Quantification of heteroplasmy

The level of m.3243A>G heteroplasmy in whole blood was quantified using Droplet Digital Polymerase chain reaction. Droplet quantification was performed using QuantaSoft Analysis Pro 1.0 software (Bio-Rad, Hercules, California), as previously described (Nielsen et al., 2025b).

### Nuclear magnetic resonance-based metabolomics

#### Sample collection and preparation

Fasting urine and serum samples were collected between 7:30 and 9:00 AM after an overnight fast of at least 8 h. Participants were instructed to abstain from their usual morning medications prior to sampling. Serum and urine samples were immediately processed and stored at −80℃ until further analysis.

#### Nuclear magnetic resonance spectroscopy

Metabolomic profiling of serum and urine samples was performed using an automated high-throughput NMR metabolomics platform (Nightingale Health Ltd., Helsinki, Finland). NMR spectroscopy was selected as it enables robust and reproducible quantification of various metabolites, such as lipoprotein subclasses and their lipid content and concentrations, apolipoproteins, and fatty acids, as well as a range of low-molecular metabolites like amino acids, ketone bodies, and metabolites related to gluconeogenesis, through an automated total-line-shape fitting protocol (Mutter et al., 2022; Soininen et al., 2015). A total of 51 metabolites in urine and 169 metabolites in serum were quantified in absolute concentrations, and three serum metabolite ratios. Detailed lists of quantified metabolites are provided in Supplementary Tables S1 and S2.

### Statistical analysis

#### Clinical characteristics

Data distribution was evaluated with quantile-quantile plots, while the homogeneity of variance was examined using the F-test. Continuous variables are presented as mean ± standard deviation for normally distributed data or median with interquartile range for non-normally distributed data. Comparisons between m.3243A>G carriers and healthy controls were performed using Student’s t-test or Wilcoxon Mann–Whitney U-test, as appropriate. Categorical variables were compared using the chi-squared test.

#### Metabolomic data analysis

Both univariate and multivariate statistical approaches were applied to explore metabolic differences between groups.

Comparisons among healthy controls, m.3243A>G carriers without diabetes, and m.3243A>G carriers with diabetes were performed using one-way analysis of variance (ANOVA) followed by Fisher’s least significant difference post hoc test. A p-value < 0.05 after adjustment for multiple testing correction was considered statistically significant.

To explore global patterns in metabolomic data, Principal Component Analysis was used as an unsupervised method to evaluate clustering and identify potential outliers. Supervised analysis was performed using sparse Partial Least Squares Discriminant Analysis (sPLS-DA) to identify metabolites contributing to group separation. Model performance was evaluated using five-fold cross-validation, and the classification error rate was calculated. Metabolites contributing to group discrimination were identified based on loading values, and heatmap were generated to visualize clustering patterns across metabolites and samples.

To evaluate the discriminatory performance of individual metabolites, exploratory univariate receiver operating characteristic (ROC) analysis was performed using the Classical Univariate ROC Curve Analysis feature within the Biomarker Analysis module of MetaboAnalyst. For urine metabolites, features were selected based on both area under the curve (AUC) ≥ 0.7 and unadjusted *p* ≤ 0.05, reflecting both discriminatory performance and statistical difference between groups. For serum metabolites, selection was based on AUC ≥ 0.7 to prioritize discriminatory performance, given the higher number of measured metabolites and the exploratory nature of the analysis. The p-values reported from this analysis are unadjusted and derived from the ROC-based framework, and were used for descriptive purposes only, rather than for determining statistical significance after multiple testing correction.

#### Machine learning analysis

To explore the classification potential of selected metabolites, several machine learning algorithms were applied, including random forest, linear support vector machine, PLS-DA, and logistic regression. Random forest models were used to classify samples into healthy controls, m.3243A>G carriers without diabetes, and carriers with diabetes, and to assess variable importance and classification error rates. Model performance was evaluated using ROC curve analysis, with calculation of the AUC and 95% confidence intervals. To assess whether classification performance exceeded random expectation, permutation testing was performed. These analyses were considered exploratory and aimed at evaluating the potential discriminatory performance of metabolite signatures.

**Fig. 1:**
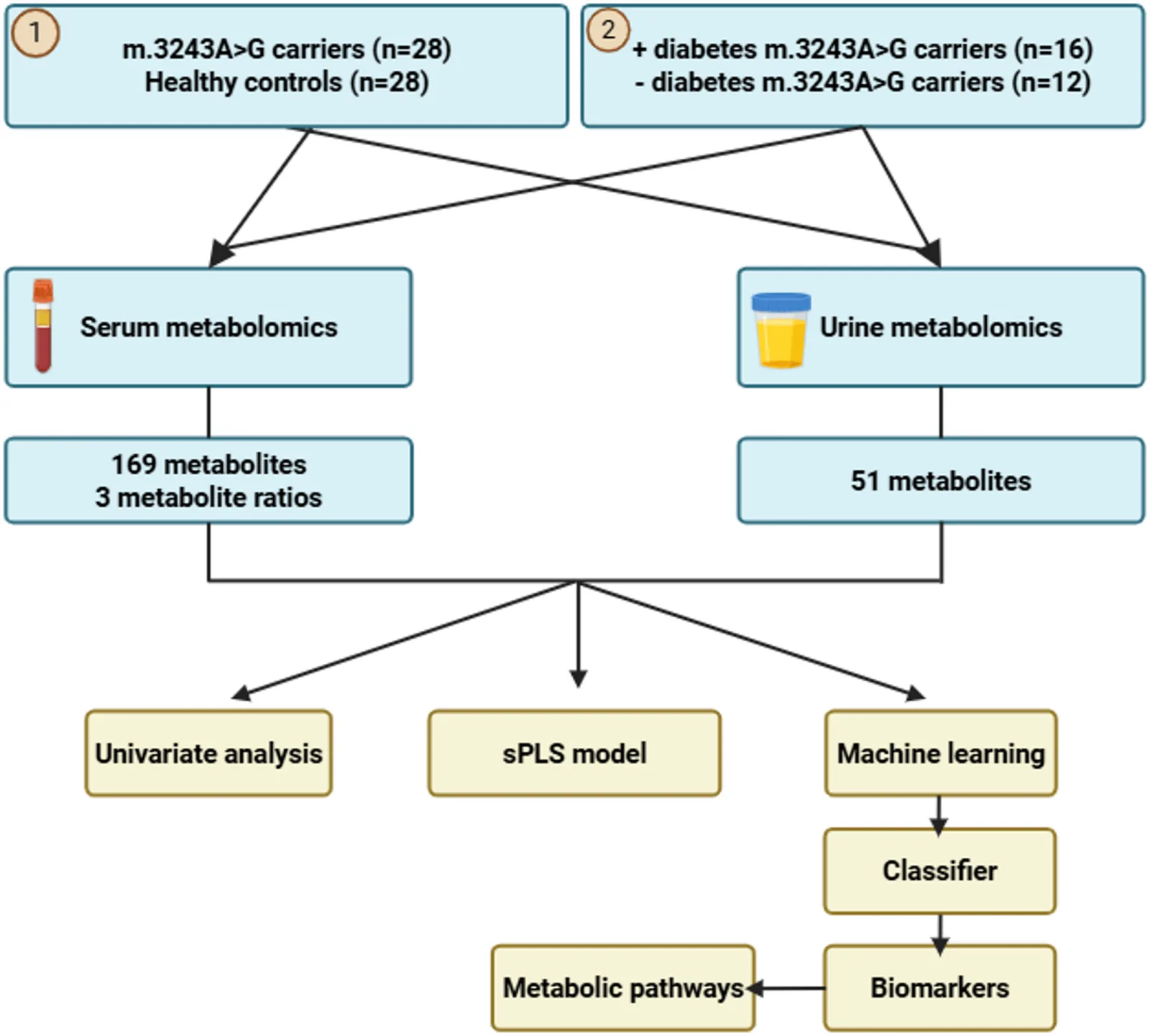
Flow chart of study. *sPLS* sparse partial least squares. Created in https://BioRender.com

A flow chart of the study is illustrated in Fig. 1. All statistical and machine learning analyses were performed using the R statistical software (version 3.5.3) with the MetaboAnalystR 4.0 package.

### Ethical approval

The study was approved by the North Denmark Region Committee on Health Research Ethics (N-20210031) and the Regional Data Protection Agency (2021−204). All procedures were conducted in accordance with the Declaration of Helsinki, and all participants provided written informed consent prior to participation. Data collection and management were facilitated using the REDCap electronic data capture tool (https://redcap.rn.dk, Vanderbilt University, Nashville, TN, USA) hosted at Region Nordjylland.

## Results

### Clinical characteristics of m.3243A>G carriers and healthy controls

A total of 28 carriers of the m.3243A>G variant from 13 different families were included in the study. Compared with healthy controls, m.3243A>G carriers had similar body mass index but significantly lower body weight and height. Biochemically, m.3243A>G carriers exhibited elevated levels of plasma triglycerides, along with reduced levels of high-density lipoprotein (HDL) compared to healthy controls. Among the m.3243A>G carriers, 57% were diagnosed with diabetes, and glycated hemoglobin (HbA1c) levels were significantly higher compared with healthy controls, who had HbA1c values within the normal range. Demographic and clinical characteristics of the groups are summarised in Table 1. The m.3243A>G carriers received various medications (Table S3), which may potentially influence their metabolic profiles.

**Table 1:** Demographic and clinical characteristics of m.3243A>G carriers and healthy controls

	m.3243A>G carriers	Controls	*p* value
Basic characteristics			
Number, n	28	28	
Sex (Female/male)	18/10	18/10	–
Age (years)	42.4 ± 12.8	42.8 ± 13.3	0.90
Physical examination			
Weight (kg)	61.4 (52.3–75.6)	70.3 (65.7–78.8)	**0.03**
Height (cm)	167.6 ± 9.0	174.1 ± 10.4	**0.01**
Body mass index (kg/m^2^)	22.1 (18.9–26.6)	23.6 (22.7–24.7)	0.27
Biochemistry			
Heteroplasmy in blood (%)	23.7 ± 13.9	–	–
HbA1c (mmol/mol)	44.0 (36.5–54.5)	33.5 (32.0–35.0)	**< 0.001**
Cholesterol (mmol/L)	4.4 ± 0.8	4.5 ± 0.8	0.81
HDL (mmol/L)	1.2 ± 0.4	1.5 ± 0.3	**0.003**
LDL (< 3.0 mmol/L)	2.5 ± 0.7	2.6 ± 0.7	0.81
Triglycerides (mmol/L)^a^	1.4 (1.1–1.7)	0.75 (0.6–1.1)	**< 0.001**
Creatinine (µmol/L)	70.9 ± 15.6	70.3 ± 13.5	0.88
ALAT (U/L)	28.0 (20.0–42.5)	23.5 (17.5–30.5)	0.08
ASAT (U/L)	26.0 (22.0–28.0)	25.5 (22.0–34.0)	0.58
Alkaline phosphatase (U/L)	77.4 ± 22.2	72.6 ± 17.0	0.38
Symptomatic assessment			
Diabetes, n (%)	16 (57%)	–	–
Ataxia, n (%)	3 (11%)	–	–
Epilepsy, n (%)	0 (0%)	–	–
Stroke-like episodes, n (%)	0 (0%)	–	–
Peripheral neuropathy, n (%)	12 (43%)	–	–
Myopathy, n (%)	18 (64%)	–	–
Hearing impairment, n (%)	19 (68%)	–	–
Cardiomyopathy, n (%)	5 (18%)	–	–
Hypertension, n (%)	10 (36%)	–	–
Non-diabetic nephropathy, n (%)	2 (7%)	–	–

Statistically significant differences are marked in bold. *HbA1c* haemoglobin A1C, *U* units, *HDL* high-density lipoproteins, *LDL* low-density lipoproteins, *ALAT* alanine aminotransferase, *ASAT* aspartate aminotransferase.

### Metabolic profile variance between m.3243A>G carriers and healthy controls

#### Urine metabolite alterations

Univariate analysis identified 16 urine metabolites with lower levels in m.3243A>G carriers compared with healthy controls [Table 2(A)]. Among these metabolites, uracil, hypoxanthine, and 1-methylnicotinamide showed the highest discriminatory performance in ROC analyses. Exploratory classification models based on these three metabolites demonstrated area under the ROC curve values ranging from 0.94 to 0.99, with cross-validated prediction accuracies between 0.81 and 0.93 across four machine-learning algorithms (linear support vector, PLS-DA, logistic regression, and random forest) [Table 2(A)].

#### Serum metabolite alterations

In serum, 25 metabolites differed between m.3243A>G carriers and healthy controls [Table 2(B)]. These alterations predominantly comprised lipoprotein subclasses and lipid composition. Specifically, m.3243A>G carriers exhibited increased triglyceride content in multiple lipoprotein subclasses and increased concentration of VLDL particles, especially small and very small. Additionally, circulating levels of lactate and pyruvate, metabolites related to glycolysis, were increased in m.3243A>G carriers. Using all 25 serum metabolites showed moderate discrimination between m.3243A>G carriers and controls with PLS-DA (AUC = 0.743, *p* = 0.038). However, other machine-learning models yielded AUC values below 0.7, indicating limited discriminatory performance for serum metabolites alone.

**Table 2:** Metabolites identified in urine (A) and serum (B) using univariate receiver operating characteristic (ROC) analysis

(A) Metabolites identified in urine								
	Direction	AUC			*p* value			Log2 FC
Uracil	↓	0.91			< 0.001			1.42
Hypoxanthine	↓	0.87			< 0.001			1.94
1-Methylnicotinamide	↓	0.82			< 0.001			1.00
Creatinine	↓	0.77			< 0.001			0.82
Pseudouridine	↓	0.76			0.001			0.75
Glycine	↓	0.75			< 0.001			1.11
Dimethylamine	↓	0.74			0.001			0.70
3-Aminoisobutyrate	↓	0.74			0.037			1.45
Glutamine	↓	0.73			0.007			0.72
Threonine	↓	0.39			0.021			0.65
Citrate	↓	0.73			0.036			0.64
Ethanolamine	↓	0.72			0.005			0.76
Pyroglutamate	↓	0.71			0.010			0.60
2-Hydroxyisobutyrate	↓	0.71			0.014			0.64
Hippurate	↓	0.71			0.028			0.94
Proline betaine	↓	0.70			0.050			1.00

Prediction models based on the top three metabolites with the highest area under the curve								
Machine learning algorithm		AUC			*p* value			Cross-validation prediction
Linear support vector		0.98 (0.93–1)			< 0.001			0.855
PLS-DA		0.97 (0.90–1)			< 0.001			0.814
Logistic regression		0.94 (0.71–1)			0.001			0.893
Random forest		0.99 (0.92–1)			< 0.001			0.928

	Direction	AUC			*p* value			Log2 FC
Triglycerides in LDL	↑	0.76			0.004			− 0.29
Triglycerides in medium LDL	↑	0.76			0.004			− 0.33
Pyruvate	↑	0.75			< 0.001			− 0.51
Degree of unsaturated fatty acids	↑	0.75			< 0.001			0.06
Triglycerides in very small VLDL	↑	0.75			0.005			− 0.36
Triglycerides in IDL	↑	0.75			0.006			− 0.28
Triglycerides in large LDL	↑	0.74			0.006			− 0.26
Triglycerides in very large HDL	↑	0.74			0.004			− 0.44
Triglycerides in small LDL	↑	0.73			0.008			− 0.41
Triglycerides in HDL	↑	0.73			0.005			−0.37
Monounsaturated fatty acids	↑	0.73			0.007			− 0.29
Lactate	↑	0.72			0.004			− 0.48
Ratio of polyunsaturated fatty acids to monounsaturated fatty acids	↓	0.72			0.002			0.21
Concentration of small VLDL particles	↑	0.72			0.018			− 0.20
Concentration of very small VLDL particles	↑	0.72			0.016			− 0.33
Total lipids in small VLDL	↑	0.72			0.015			− 0.32
Concentration of VLDL particles	↑	0.71			0.013			− 0.30
Cholesteryl esters in small VLDL	↑	0.71			0.026			− 0.27
Total lipids in very small VLDL	↑	0.71			0.018			− 0.21
Phospholipids in very small VLDL	↑	0.71			0.021			− 0.23
Triglycerides in small HDL	↑	0.71			0.009			− 0.36
Cholesterol in small VLDL	↑	0.70			0.032			− 0.25
Phospholipids in small VLDL	↑	0.70			0.025			− 0.26
Triglycerides in medium HDL	↑	0.70			0.009			− 0.37
Acetate	↓	0.70			0.006			0.72

Area under the curve (AUC) indicates discriminatory performance. *p*-values are derived from ROC-based analysis and are not adjusted for multiple testing

Values in parentheses represent the 95% confidence intervals. ↑ indicates higher metabolite levels in m.3243A>G carriers compared with healthy controls. ↓ indicates lower metabolite levels in m.3243A>G carriers compared with healthy controls. *AUC* area under the curve, *FC* fold change, *PLS-DA* partial least squares-descriminant analysis, *LDL* low-density lipoproteins, *VLDL* very-low-density lipoproteins, *IDL* intermediate density lipoproteins, *HDL* high-density lipoproteins

^a^Using all 25 serum metabolites m.3243A>G carriers could be differentiated from healthy controls with Partial Least Squares-Descriminant Analysis (area under the curve (AUC) = 0.743, *p* = 0.038). However, with linear support vector, logistic regression or random forest machine learning models, the 25 metabolites could not differentiate between the two groups (AUC < 0.7)

#### Multivariate analysis

**Fig. 2:**
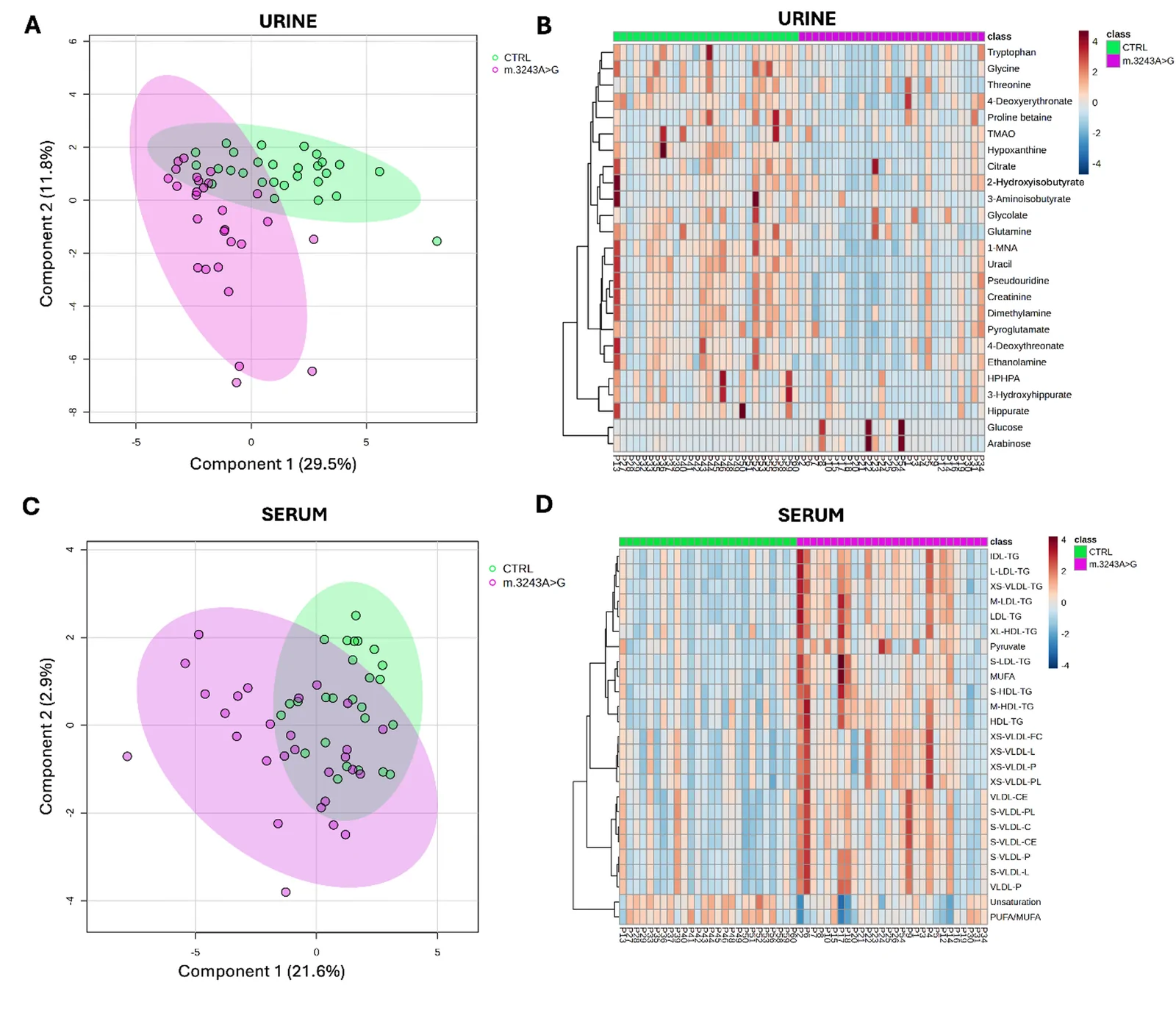
Discriminative models distinguishing m.3243A>G carriers from healthy controls based on urine (**A** and **B**) and serum (**C** and **D**) metabolic profiles. Panels **A** and **C** show sparse Partial Least Squares Discriminant Analysis (sPLS-DA) plots, illustrating separation between m.3243A>G carriers (purple) and healthy controls (green). Panels **B** and **D** present hierarchical clustered heatmaps of the top 25 differentially abundant metabolites. Hierarchical clustering was applied to both metabolites (rows) and study participants (columns), with clustering patterns visualized through dendrograms. Abbreviations: CTRL – control. For abbreviations of the metabolites, see Tables S1 and S2

To evaluate global metabolic differences between groups, metabolic profiles in urine and serum were analyzed using sPLS-DA. For urine samples, the sPLS-DA model showed separation between m.3243A>G carriers and healthy controls with a classification accuracy of 80.72%, while the model explained 41.3% of the variance across the first two components (Fig. 2A). For serum samples, separation between the groups was less pronounced, with a classification accuracy of 68.6% and 24.5% of the variance explained by the first two components (Fig. 2C). Hierarchical clustering heat maps of the 25 most discriminating metabolites further illustrated metabolic differences between carriers and controls in urine and serum (Fig. 2B, D).

### Metabolic profile differences between m.3243A>G carriers with and without diabetes

Among the 28 m.3243A>G carriers, 16 individuals were diagnosed with diabetes. Carriers with diabetes were older than carriers without diabetes (age: 47.1 ± 11.2 vs. 36.1 ± 12.4 years, *p* = 0.02) and had, as expected, higher HbA1c levels. Moreover, they exhibited a higher prevalence of all assessed clinical symptoms compared to carriers without diabetes, except for nephropathy. However, only the frequencies of myopathy, cardiomyopathy, and hearing impairment were significantly increased in m.3243A>G carriers with diabetes (Supplementary Table S4).

#### Urine metabolite alterations

**Fig. 3:**
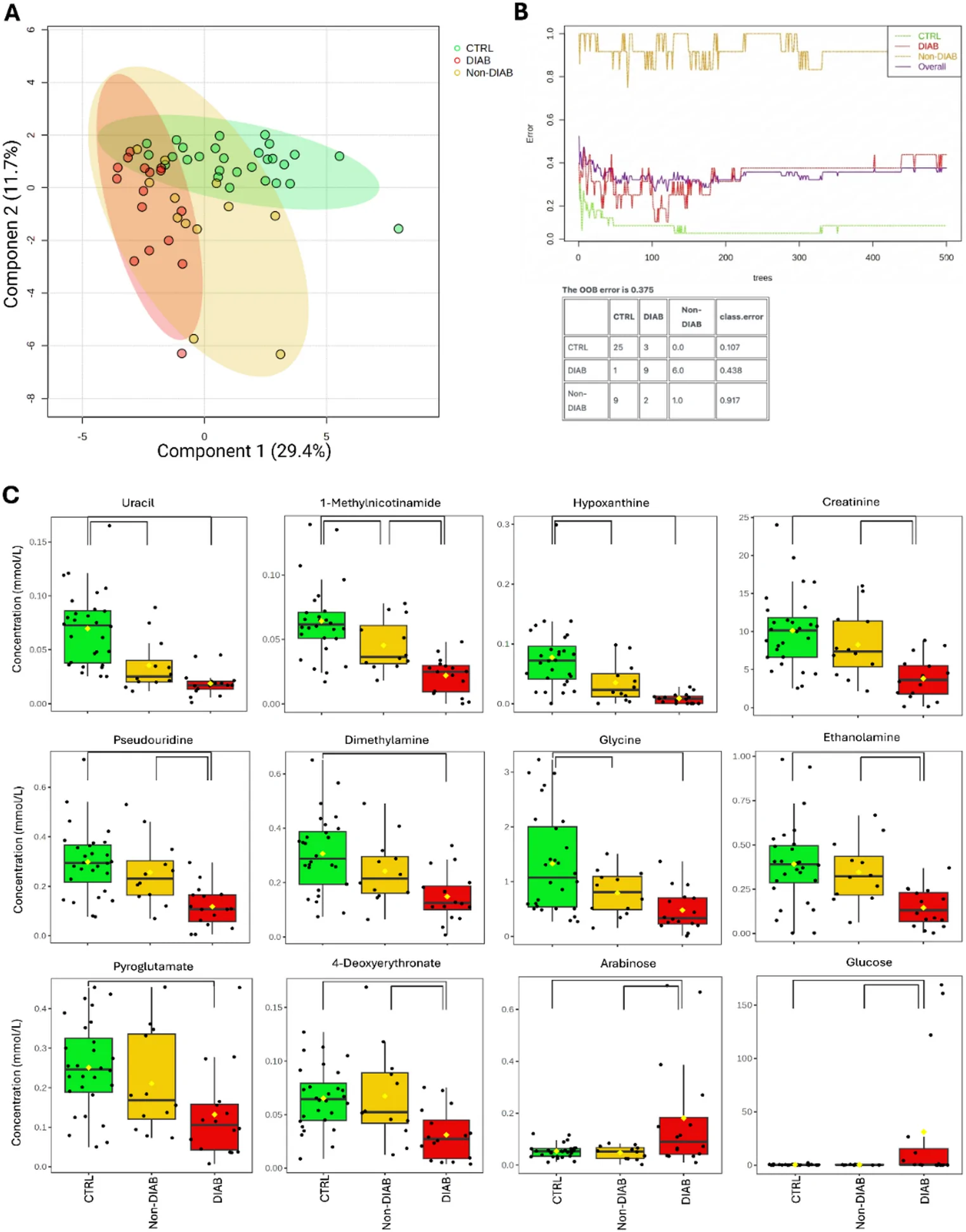
Sparse Partial Least Squares Discriminant Analysis (sPLS-DA) (**A**) and Random Forest classification of urine samples from healthy control (green), m.3243A>G carriers without diabetes (yellow), and m.3243A>G carriers with diabetes (red) (**B**). Twelve urine metabolites were identified as significantly different among the three groups using Fisher’s Least Significant Difference analysis (**C**). Boxes represent medians and interquartile ranges, while the yellow diamonds are means. Whiskers are the range of non-outlier values (up to 1.5 x interquartile range). Significant differences with *p* < 0.05 in post hoc tests between groups are illustrated as brackets between groups. Abbreviations: OOB - out-of-bag score; CTRL - healthy controls; Non-DIAB - m.3243A>G carriers without diabetes; DIAB – m.3243A>G carriers with diabetes

An sPLS-DA model compared global metabolic differences between m.3243A>G carriers with diabetes, carriers without diabetes and healthy controls, while a random forest model was applied for group classification (Fig. 3A, B).

ANOVA comparing healthy controls, carriers without diabetes, and carriers with diabetes identified 12 urine metabolites that differed significantly among the three groups (Fig. 3C). For 10 of the 12 metabolites, the highest concentrations were observed in healthy controls, and the lowest in m.3243A>G carriers with diabetes. Arabinose and glucose followed a different pattern. Post hoc comparison between m.3243A>G carriers with and without diabetes revealed seven significant altered urine metabolites, including pseudouridine, creatinine, ethanolamine, deoxyerythronate, arabinose, 1-methylnicotinamide, and glucose) (Supplementary Table S5).

#### Serum metabolite alterations

After adjustment for multiple testing, no serum metabolites differed significantly between the three groups. However, before correcting for multiple comparisons, the total branched-chain amino acids (BCAA) level and valine were significantly different between groups (ANOVA *p* = 0.029 and *p* = 0.022, respectively), whereas leucine and isoleucine showed similar trends but did not reach statistical significance (ANOVA *p* = 0.051 and *p* = 0.055, respectively).

#### Univariate and multivariate analysis comparing m.3243A>G carriers with and without diabetes

**Fig. 4:**
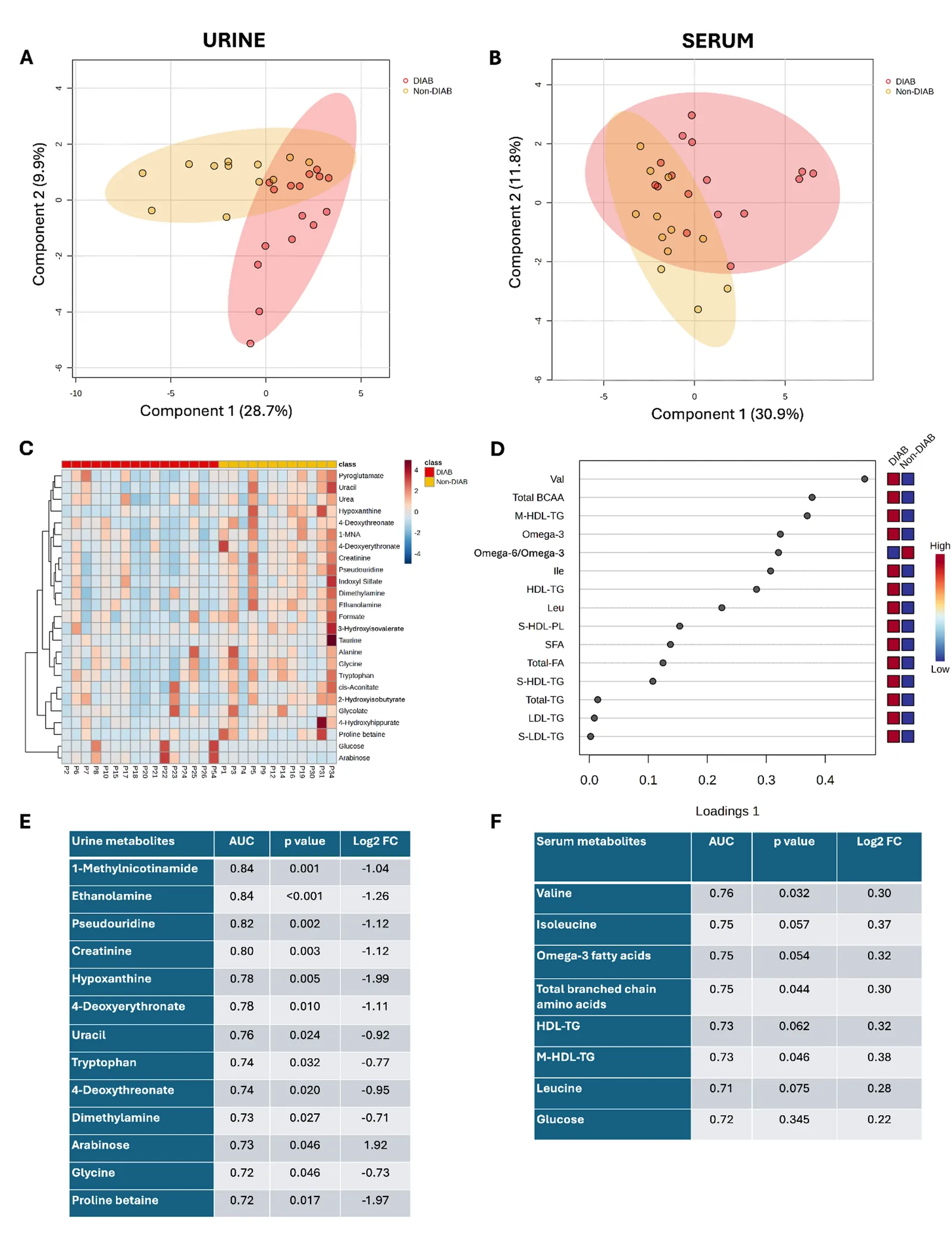
Metabolomics analysis distinguishing m.3243A>G carriers with and without diabetes. Panels **A**,** C**, and **E** show urine metabolomic analysis, whereas panels **B**,** D**, and **F** show serum metabolomic analysis. Panels **A** and **B** display sparse Partial Least Squares Discriminant Analysis (sPLS-DA) score plots comparing m.3243A>G carriers without diabetes (yellow) and carriers with diabetes (red). Panel **C** represents hierarchical clustering analysis of differentially abundant metabolites in urine samples, while panel **D** presents a loading plot based on serum samples. Panels **E** and **F** show the metabolites contributing to discrimination between m.3243A>G carriers with and without diabetes in urine and serum samples, respectively. For abbreviations of the metabolites, see Supplementary Tables S1 and S2

To further explore metabolic differences between m.3243A>G carriers with and without diabetes, sPLS-DA models were applied to urine and serum metabolomic datasets (Fig. 4A, B). The classification accuracy of the sPLS-DA model was 67.0% for urine samples and 54.3% for serum samples, indicating modest separation between the two groups.

Using univariate analysis, a total of 13 urine metabolites and eight serum metabolites were found to differ between m.3243A>G carriers with and without diabetes (Fig. 4E, F). Exploratory machine learning models based on the three most discriminating urine metabolites achieved AUC values ranging from 0.76 to 0.84, with cross-validation prediction accuracies between 0.679 and 0.722 (Supplementary Table S6A). In serum, m.3243A>G carriers with diabetes had elevated levels of triglycerides within HDL particles, particularly medium-sized HDL, along with increased levels of omega-3 fatty acids, branched-chain amino acids (isoleucine, leucine, and valine), and glucose (Fig. 4F). Machine learning models based on serum metabolites could not discriminate between the groups (AUC < 0.7).

### Additional group-wise comparisons

Additional comparisons between healthy controls and m.3243A>G carriers with diabetes, as well as between healthy controls and m.3243A>G carriers without diabetes, are presented in the supplementary material (Fig. S1, S2, and Table S6B, S7). In brief, five serum metabolites (the degree of unsaturated fatty acids, acetate, pyruvate, glutamine, and acetone) and four in urine (uracil, glutamine, hypoxanthine, and cis-aconitate) differed between healthy controls and m.3243A>G carriers without diabetes, while 44 serum metabolites and 18 urine metabolites differed between controls and carriers with diabetes.

## Discussion

Comprehensive metabolic profiling of 28 individuals carrying the pathogenic mitochondrial variant m.3243A>G demonstrates a distinct dysregulation of lipoprotein metabolism. In addition, exploratory analyses indicated that a combination of three urine metabolites showed discriminatory potential between m.3243A>G carriers and healthy controls. Finally, we observed higher circulating levels of branched-chain amino acids in m.3243A>G carriers with diabetes compared with carriers without diabetes.

### Dysregulated lipoprotein metabolism in m.3243A>G carriers

Lipoprotein profiling demonstrated elevated triglyceride levels across multiple lipoprotein subclasses, together with increased concentrations of circulating VLDL particles in m.3243A>G carriers. Increased triglyceride-rich lipoproteins have been associated with an increased risk of developing type 2 diabetes (Ahola-Olli et al., 2019; Bragg et al., 2022; Seah et al., 2022). These large-scale cohort studies, however, reported additional risk factors not observed in our cohort, including elevated apolipoprotein B levels and increased VLDL particle size. In type 2 diabetes, the proposed mechanisms of increased triglyceride levels include insulin resistance in adipose tissue, resulting in an enhanced flux of free fatty acids to the liver, enhanced hepatic de novo lipogenesis, and elevated hepatic uptake of triglyceride-rich lipoprotein remnant particles (Vergès, 2015). Similar metabolic mechanisms may contribute to increased triglyceride levels in m.3243A>G carriers. In contrast to the obesity-related type 2 diabetes, the m.3243A>G carriers are typically non-obese and do not exhibit ectopic fat accumulation in organs such as the liver, pancreas, and skeletal muscle (Lindroos et al., 2011; Nielsen et al., 2025a). However, in m.3243A>G carriers, severe muscle atrophy may be replaced by adipose tissue infiltration (Kärppä et al., 2004). Moreover, VLDL assembly and secretion are stimulated by high triglyceride levels, which may account for the increased concentration of VLDL particles. Alternatively, this finding may reflect the underlying insulin resistance, as impaired insulin signaling diminishes the suppression of VLDL formation (Borén et al., 2022). Another possible explanation is impaired mitochondrial fatty acid β-oxidation, as indicated by altered urine metabolites involved in fatty acid metabolism in a previous metabolomics study of m.3243A>G carriers with the severe phenotype mitochondrial encephalomyopathy, lactic acidosis, and stroke-like episodes (MELAS) (Esterhuizen et al., 2019).

From a clinical perspective, triglyceride-rich lipoproteins, altered lipoprotein particle concentration, and size have been associated with cardiovascular disease in both type 1 and type 2 diabetes, particularly atherosclerosis, myocardial infarction, and ischemic stroke (Baratta et al., 2023; Borén et al., 2022; Mäkinen et al., 2013; Nordestgaard and Varbo, 2014). Notably, prospective analysis in individuals with type 2 diabetes within the UK Biobank demonstrated associations between elevated triglyceride content of specific lipoprotein subclasses and increased all-cause mortality, rather than being confined to cardiovascular mortality (Li et al., 2023). Cardiovascular mortality represents a major cause of premature mortality in m.3243A>G carriers, though it is usually caused by left ventricular hypertrophy or conduction defects rather than atherosclerosis (Barends et al., 2016; Malfatti et al., 2013; Papadopoulos et al., 2020). Nevertheless, a pro-atherogenic phenotype has been reported in a study using induced pluripotent stem cell-derived endothelial cells from m.3243A>G carriers with high (> 80%) heteroplasmy levels (Pek et al., 2019). These cells exhibited elevated levels of oxidized low-density lipoprotein, suggesting a potential intrinsic susceptibility to atherogenesis. Collectively, the mechanisms underlying dysregulated lipoprotein metabolism and its clinical consequences for cardiovascular disease and mortality in m.3243A>G carriers remain unknown.

### Metabolic pathway alterations in m.3243A>G carriers

Beyond lipoprotein alterations, our findings indicate that multiple metabolic pathways are disrupted in m.3243A>G carriers. First, alterations in metabolites linked to glycolysis and the tricarboxylic acid cycle were observed, including elevated circulating pyruvate and lactate levels, reduced acetate concentration, and decreased urinary levels of citrate and glutamine. Other studies have found lower levels of other tricarboxylic acid cycle intermediates, including fumaric, malic, and succinic acid, being characteristic of m.3243A>G carriers (Cao et al., 2025; Esterhuizen et al., 2021) together with elevated levels of pyruvate and lactate, considered classic biomarkers for mitochondrial disease (Hubens et al., 2022). Moreover, 1-methylnicotinamide is involved in the NAD^+^ homeostasis and has previously been reported to be decreased in m.3243A>G carriers (Hall et al., 2015). Second, we found lower levels of urine pyroglutamate, indicative of dysregulated antioxidative defence through the glutathione metabolism, consistent with previous findings of disturbed glutathione metabolism in m.3243A>G carriers (Esterhuizen et al., 2021; Hall et al., 2015). Third, metabolites involved in one-carbon metabolism and the methylation cycle (reflected by decreased urine glycine and threonine) are pathways previously discovered to be altered in mitochondrial diseases (Buzkova et al., 2018; Esterhuizen et al., 2021). Fourth, purine (lower levels of urine hypoxanthine) and pyrimidine metabolism (lower levels of uracil and glutamine) as part of the nucleotide metabolism were perturbed in m.3243A>G carriers. Uracil is a pyrimidine, which has previously been reported to be elevated in the urine of children with respiratory chain deficiencies (Reinecke, 2012). Another study identified elevated levels of xanthine in MELAS patients, which supports our finding of increased hypoxanthine, indicative of affected purine metabolism (Esterhuizen et al., 2019). Collectively, these metabolic perturbations support the concept that impaired oxidative phosphorylation affects multiple interconnected metabolic pathways. However, some urinary metabolite differences, including glucose and arabinose, may reflect diabetes-related metabolic disturbances rather than mitochondrial dysfunction.

### Urinary metabolite signatures distinguishing m.3243A>G carriers from healthy controls

In exploratory analyses, urinary metabolite profiles showed stronger discriminatory performance than serum metabolites for distinguishing m.3243A>G carriers from healthy controls. A combination of urine uracil, hypoxanthine, and 1-methylnicotinamide demonstrated high classification performance in machine-learning models. Metabolomic profiling, influenced by both platform and tissue type, has revealed distinct biochemical signatures associated with the m.3243A>G variant across clinical phenotypes. In urine, Esterhuizen et al. identified 36 urine metabolites distinguishing individuals with the severe MELAS phenotype from controls (Esterhuizen et al., 2019), while a complementary multi-platform study identified consistent alterations in 2-hydroxyglutaric acid, glycolic acid, and 4-pentenoic acid across all m.3243A>G phenotypes (Esterhuizen et al., 2021). In a blood-based metabolomics study, Buzkova et al. reported strong diagnostic performance for mitochondrial disease achieving an AUC of 0.94 using a four-metabolite panel comprising sorbitol, alanine, myo-inositol, and cystathionine detected by mass spectrometry (Buzkova et al., 2018). In contrast, serum metabolites in our study showed limited discriminatory performance, which may reflect differences in analytical platforms, cohort composition, or the metabolites detected by NMR-based metabolomics compared with mass spectrometry. Although the urinary metabolite panel identified here demonstrated promising discriminatory performance, these findings should be interpreted cautiously and require validation in independent cohorts before potential clinical applications such as disease monitoring or treatment response assessment may be considered.

### Branched-chain amino acids and diabetes in m.3243A>G carriers

BCAAs, including valine, leucine, and isoleucine, are essential amino acids involved in protein synthesis and energy metabolism. Elevated BCAA concentrations have previously been reported in both urine and blood in individuals with primary mitochondrial disease (Clarke et al., 2013; El-Hattab et al., 2012; Esterhuizen et al., 2019). In contrast to Esterhuizen et al., who reported impaired BCAA catabolism across all m.3243A>G phenotypes, including those without diabetes (Esterhuizen et al., 2021), our findings suggest that serum BCAA levels were higher in m.3243A>G carriers with diabetes compared with carriers without diabetes. However, m.3243A>G carriers with diabetes were not matched for sex, were older, and exhibited more severe clinical manifestations, including a higher prevalence of myopathy, cardiomyopathy, and hearing impairment. These differences may reflect sex-specific metabolic patterns or a more advanced or severe disease phenotype and could confound the observed metabolic differences. Elevated BCAA levels have consistently been associated with insulin resistance and an increased risk of type 2 diabetes in population studies (Morze et al., 2022; Wang et al., 2011; Würtz et al., 2013). Whether BCAAs directly contribute to or simply represent biomarkers of insulin resistance in type 2 diabetes remains unresolved (Lynch and Adams, 2014). Nevertheless, elevated BCAAs could potentially reflect an underlying insulin resistance in m.3243A>G carriers with diabetes, similar to observations in type 2 diabetes.

### Strengths and limitations

This study has several strengths. First, all samples were collected under a standardized fasting protocol, minimizing metabolic variability related to recent food intake. Second, m.3243A>G carriers and controls were closely matched for age and sex and had similar body mass index. Third, to our knowledge, this study represents the first investigation combining serum and urine metabolomics with detailed lipoprotein subclass profiling in individuals carrying the m.3243A>G variant. Limitations include a relatively small sample size, reflecting the rarity of m.3243A>G-associated mitochondrial disease, which may affect the statistical power and the generalizability of our findings. Some participants originated from the same families, reflecting the hereditary nature and rarity of m.3243A>G-associated disease. Familial relatedness may contribute to shared metabolic characteristics and should therefore be considered when interpreting the findings. Urinary metabolites were analyzed using absolute concentrations rather than creatinine normalization, as creatinine metabolism may itself be altered in mitochondrial diseases (Esterhuizen et al., 2017; Hall et al., 2015). Although the use of fasting morning spot urine samples reduces variability related to hydration status (Li et al., 2022), residual variation in urine concentration cannot be excluded. Future studies, including larger cohorts and parallel evaluation of multiple normalization strategies for individuals with systemic metabolic conditions, may help further assess the robustness of urinary metabolite findings. Although participants abstained from taking their morning medications before sample collection, medication use may still have influenced urinary metabolite profiles. In particular, diabetes, hypertension, nephropathy, and medications, including antidiabetic, antihypertensive, and lipid-lowering therapies, may influence metabolite concentrations and lipoprotein profiles (Gianazza et al., 2023). In line with previous metabolomics studies on mitochondrial diseases (Buzkova et al., 2018; Esterhuizen et al., 2021; Hall et al., 2015), relevant clinical characteristics and medication were documented. However, given the limited sample size inherent to research in rare diseases, the study was not powered to perform stratified or fully adjusted analyses, and residual confounding cannot be excluded. Finally, the cross-sectional design precludes conclusions regarding causal relationships or the long-term clinical implications of the observed metabolic alterations.

## Conclusion

We identified a distinctive serum lipoprotein signature in m.3243A>G carriers characterized by elevated triglyceride content across multiple lipoprotein subclasses and a higher concentration, together with altered composition of VLDL particles. Collectively, this indicates a dysregulation of lipoprotein metabolism in individuals with the m.3243A>G mitochondrial impairment of OXPHOS. Longitudinal prospective studies in larger cohorts will be essential to validate these metabolic signatures and to clarify their potential relevance for the m.3243A>G phenotype and risk of premature mortality.

## Supplementary Information

Below is the link to the electronic supplementary material.

Supplementary Material 1

## Data Availability

The datasets produced and/or analyzed in this study can be obtained from the corresponding author upon reasonable request.
