# Supplementary material for: Metabolomic signature reveals dysregulated lipoprotein profile in m.3243A>G carriers: a case-control study
Source: Metabolomics. 2026 Jul 3;22(4):118. doi: 10.1007/s11306-026-02503-8 (PMC13331837; doi:10.1007/s11306-026-02503-8)
Supplement: Supplementary file 1 — Supplementary Material 1 [file 11306_2026_2503_MOESM1_ESM.docx]

**Supplementary Material**

**Metabolomic signature reveals dysregulated lipoprotein profile in m.3243A>G carriers: a Case-Control Study**

*Metabolomics*

Simone Rask Nielsen^1,2^. Hien Thi Thu Nguyen^2,3^. Malene Pontoppidan Stoico^2,3^. Christina Brock^2,4^. Kurt Højlund^5,6^. Inge Søkilde Pedersen*^2,3^. Anja Lisbeth Frederiksen*^1,2,6,7^

^1^Department of Clinical Genetics, Aalborg University Hospital, Aalborg, Denmark | ^2^Department of Clinical Medicine, Aalborg University, Aalborg, Denmark | ^3^Department of Molecular Diagnostics, Aalborg University Hospital, Aalborg, Denmark | ^4^Mech-Sense, Department of Gastroenterology and Hepatology, Aalborg University Hospital, Aalborg, Denmark | ^5^Steno Diabetes Center Odense, Odense University Hospital, Odense, Denmark | ^6^Department of Clinical Research, University of Southern Denmark, Odense, Denmark | ^7^Department of Clinical Genetics, Odense University Hospital, Odense, Denmark |

*Shared last co-authorship: Anja Lisbeth Frederiksen and Inge Søkilde

**Corresponding author:**

Simone Rask Nielsen, s.rask@rn.dk, Dept. of Clinical Genetics, Aalborg University Hospital, Aalborg, Denmark & Dept. of Clinical Medicine, Aalborg University, Aalborg, Denmark.


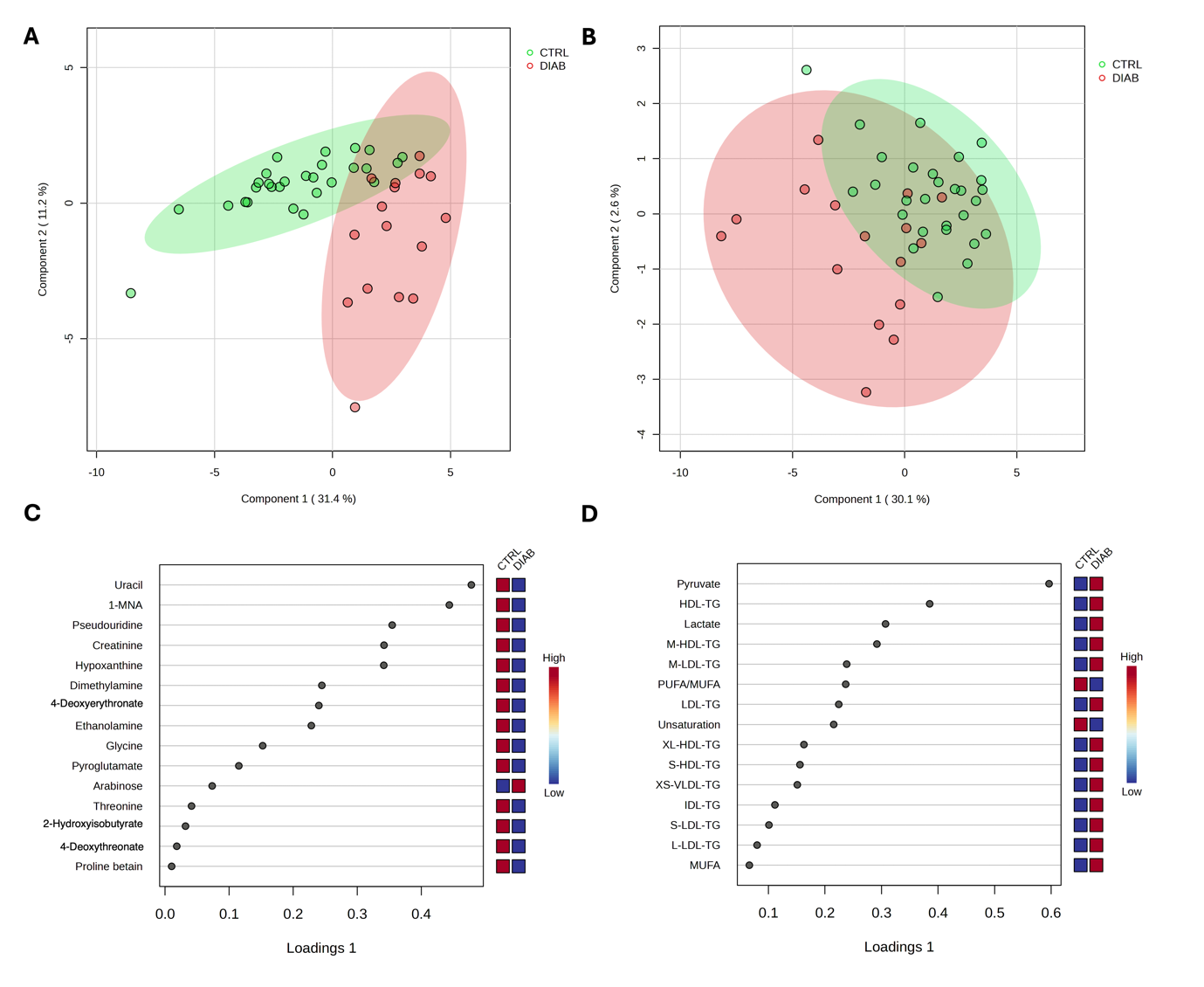


**Fig. S1** Sparse Partial Least Squares Discriminant Analysis (sPLS-DA) score plots and corresponding loading plots of differentially abundant metabolites in urine (**A** and **C**) and serum (**B** and **D**) samples from healthy controls (green) and m.3243A>G carriers with diabetes (red). The sPLS-DA model based on urine metabolites effectively discriminated between m.3243A>G carriers without diabetes and healthy controls, achieving an accuracy of 89.37%. In contrast, the model based on serum metabolites showed limited discriminatory power, with an accuracy of 66.36%.

Abbreviations: CTRL – controls; DIAB – m.3243A>G carriers with diabetes. For abbreviations of the metabolites, see Table S1 and S2.


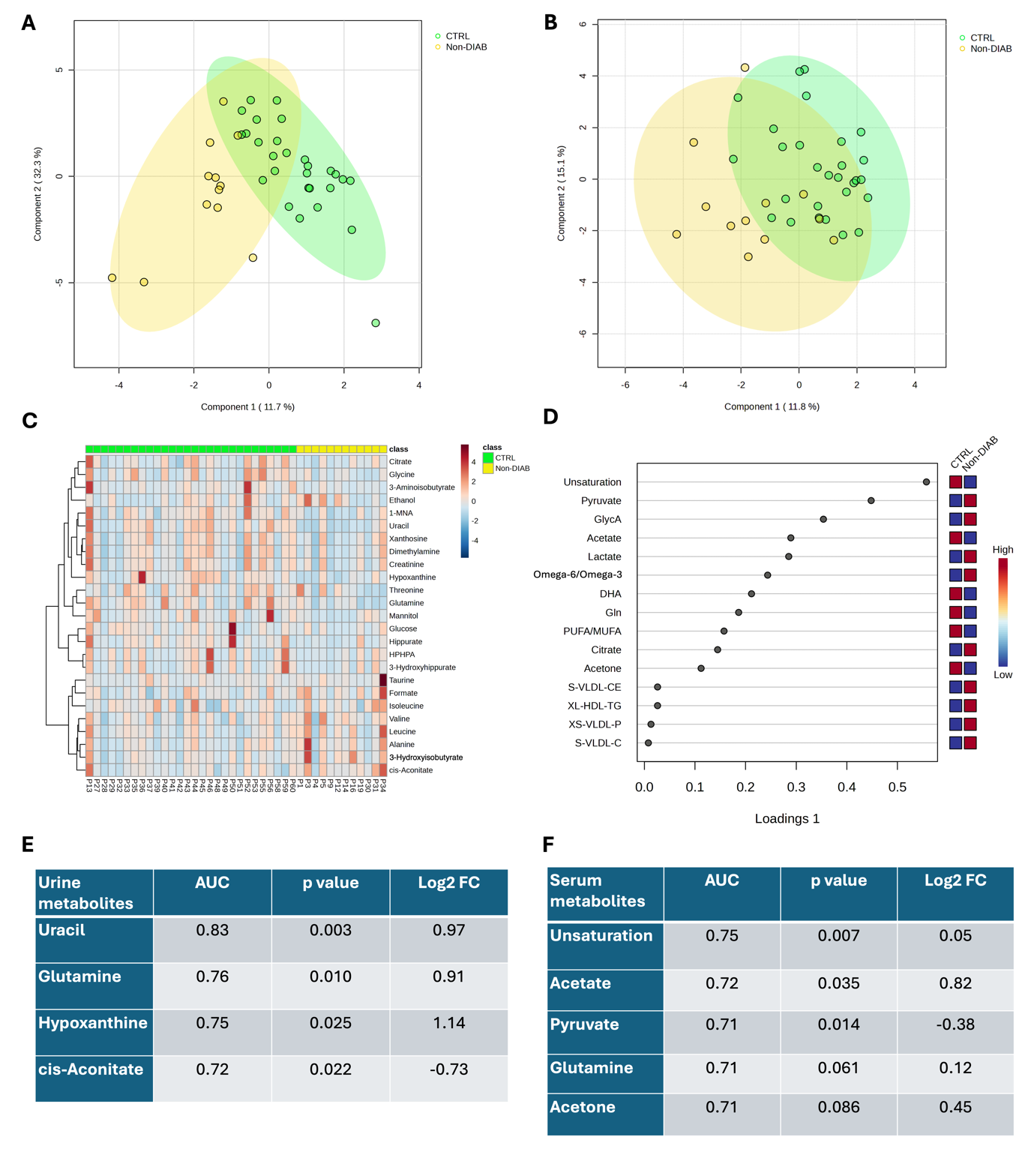


**Fig. S2** Sparse Partial Least Squares Discriminant Analysis (sPLS-DA) score plots, hierarchical clustering heatmap or corresponding loading plots of differentially abundant metabolites, and metabolites differing between groups in urine (**A, C**, and **E**) and serum (**B, D,** and **F**) samples comparing healthy controls (green) and m.3243A>G carriers without diabetes (yellow). The sPLS-DA model based on urine metabolites effectively discriminating between carriers without diabetes and healthy controls, achieving an accuracy of 80%. In contrast, the model based on serum metabolites showed limited discriminatory power, with an accuracy of 66%.

Machine learning models using the top four urine metabolites were able to distinguish between groups with AUC 0.86-0.95 and coefficient of variation prediction 0.78-0.90 (Supplementary Table S6B). Machine learning models using serum metabolites were not able to differentiate between groups with AUC<0.7.

Abbreviations: CTRL – controls; Non-DIAB – m.3243A>G carriers without diabetes; AUC area under the curve; FC – fold change. For abbreviations of the metabolites, see Table S1 and S2.

**Table S1** List of 169 metabolites and three metabolite ratios identified by serum metabolomics

| **No** | **Serum metabolites** | **Short name** | **Group** | |
| --- | --- | --- | --- | --- |
| 1 | Total cholesterol | Total_C | Cholesterol |  |
| 2 | Total cholesterol minus HDL-cholesterol | non_HDL_C | Cholesterol |  |
| 3 | Remnant cholesterol (non-HDL, non-LDL -cholesterol) | Remnant_C | Cholesterol |  |
| 4 | VLDL cholesterol | VLDL_C | Cholesterol |  |
| 5 | Clinical LDL cholesterol | Clinical_LDL_C | Cholesterol |  |
| 6 | LDL cholesterol | LDL_C | Cholesterol |  |
| 7 | HDL cholesterol | HDL_C | Cholesterol |  |
| 8 | Total triglycerides | Total_TG | Glycerides and phospholipids |  |
| 9 | Triglycerides in VLDL | VLDL_TG | Glycerides and phospholipids |  |
| 10 | Triglycerides in LDL | LDL_TG | Glycerides and phospholipids |  |
| 11 | Triglycerides in HDL | HDL_TG | Glycerides and phospholipids |  |
| 12 | Total phospholipids in lipoprotein particles | Total_PL | Glycerides and phospholipids |  |
| 13 | Phospholipids in VLDL | VLDL_PL | Glycerides and phospholipids |  |
| 14 | Phospholipids in LDL | LDL_PL | Glycerides and phospholipids |  |
| 15 | Phospholipids in HDL | HDL_PL | Glycerides and phospholipids |  |
| 16 | Total esterified cholesterol | Total_CE | Cholesterol |  |
| 17 | Cholesteryl esters in VLDL | VLDL_CE | Cholesterol |  |
| 18 | Cholesteryl esters in LDL | LDL_CE | Cholesterol |  |
| 19 | Cholesteryl esters in HDL | HDL_CE | Cholesterol |  |
| 20 | Total free cholesterol | Total_FC | Cholesterol |  |
| 21 | Free cholesterol in VLDL | VLDL_FC | Cholesterol |  |
| 22 | Free cholesterol in LDL | LDL_FC | Cholesterol |  |
| 23 | Free cholesterol in HDL | HDL_FC | Cholesterol |  |
| 24 | Total lipids in lipoprotein particles | Total_L | Total lipids |  |
| 25 | Total lipids in VLDL | VLDL_L | Total lipids |  |
| 26 | Total lipids in LDL | LDL_L | Total lipids |  |
| 27 | Total lipids in HDL | HDL_L | Total lipids |  |
| 28 | Total concentration of lipoprotein particles | Total_P | Lipoprotein particle concentrations |  |
| 29 | Concentration of VLDL particles | VLDL_P | Lipoprotein particle concentrations |  |
| 30 | Concentration of LDL particles | LDL_P | Lipoprotein particle concentrations |  |
| 31 | Concentration of HDL particles | HDL_P | Lipoprotein particle concentrations |  |
| 32 | Average diameter for VLDL particles | VLDL_size | Lipoprotein particle sizes |  |
| 33 | Average diameter for LDL particles | LDL_size | Lipoprotein particle sizes |  |
| 34 | Average diameter for HDL particles | HDL_size | Lipoprotein particle sizes |  |
| 35 | Phosphoglycerides | Phosphoglyc | Glycerides and phospholipids |  |
| 36 | Total cholines | Cholines | Glycerides and phospholipids |  |
| 37 | Phosphatidylcholines | Phosphatidylc | Glycerides and phospholipids |  |
| 38 | Sphingomyelins | Sphingomyelins | Glycerides and phospholipids |  |
| 39 | Apolipoprotein B | ApoB | Apolipoproteins |  |
| 40 | Apolipoprotein A1 | ApoA1 | Apolipoproteins |  |
| 41 | Ratio of apolipoprotein B to apolipoprotein A1 | ApoB_by_ApoA1 | Apolipoproteins |  |
| 42 | Total fatty acids | Total_FA | Fatty acids |  |
| 43 | Degree of unsaturation | Unsaturation | Fatty acids |  |
| 44 | Omega-3 fatty acids | Omega_3 | Fatty acids |  |
| 45 | Omega-6 fatty acids | Omega_6 | Fatty acids |  |
| 46 | Polyunsaturated fatty acids | PUFA | Fatty acids |  |
| 47 | Monounsaturated fatty acids | MUFA | Fatty acids |  |
| 48 | Saturated fatty acids | SFA | Fatty acids |  |
| 49 | Linoleic acid | LA | Fatty acids |  |
| 50 | Docosahexaenoic acid | DHA | Fatty acids |  |
| 51 | Ratio of polyunsaturated fatty acids to monounsaturated fatty acids | PUFA_by_MUFA | Fatty acids |  |
| 52 | Ratio of omega-6 fatty acids to omega-3 fatty acids | Omega_6_by_Omega_3 | Fatty acids |  |
| 53 | Alanine | Ala | Amino acids |  |
| 54 | Glutamine | Gln | Amino acids |  |
| 55 | Glycine | Gly | Amino acids |  |
| 56 | Histidine | His | Amino acids |  |
| 57 | Total concentration of branched-chain amino acids (leucine + isoleucine + valine) | Total_BCAA | Amino acids |  |
| 58 | Isoleucine | Ile | Amino acids |  |
| 59 | Leucine | Leu | Amino acids |  |
| 60 | Valine | Val | Amino acids |  |
| 61 | Phenylalanine | Phe | Amino acids |  |
| 62 | Tyrosine | Tyr | Amino acids |  |
| 63 | Glucose | Glucose | Glycolysis related metabolites |  |
| 64 | Lactate | Lactate | Glycolysis related metabolites |  |
| 65 | Pyruvate | Pyruvate | Glycolysis related metabolites |  |
| 66 | Citrate | Citrate | Glycolysis related metabolites |  |
| 67 | Glycerol | Glycerol | Glycolysis related metabolites |  |
| 68 | 3-Hydroxybutyrate | bOHbutyrate | Ketone bodies |  |
| 69 | Acetate | Acetate | Ketone bodies |  |
| 70 | Acetoacetate | Acetoacetate | Ketone bodies |  |
| 71 | Acetone | Acetone | Ketone bodies |  |
| 72 | Creatinine | Creatinine | Fluid balance |  |
| 73 | Albumin | Albumin | Fluid balance |  |
| 74 | Glycoprotein acetyls | GlycA | Inflammation |  |
| 75 | Concentration of chylomicrons and extremely large VLDL particles | XXL_VLDL_P | Lipoprotein subclasses |  |
| 76 | Total lipids in chylomicrons and extremely large VLDL | XXL_VLDL_L | Lipoprotein subclasses |  |
| 77 | Phospholipids in chylomicrons and extremely large VLDL | XXL_VLDL_PL | Lipoprotein subclasses |  |
| 78 | Cholesterol in chylomicrons and extremely large VLDL | XXL_VLDL_C | Lipoprotein subclasses |  |
| 79 | Cholesteryl esters in chylomicrons and extremely large VLDL | XXL_VLDL_CE | Lipoprotein subclasses |  |
| 80 | Free cholesterol in chylomicrons and extremely large VLDL | XXL_VLDL_FC | Lipoprotein subclasses |  |
| 81 | Triglycerides in chylomicrons and extremely large VLDL | XXL_VLDL_TG | Lipoprotein subclasses |  |
| 82 | Concentration of very large VLDL particles | XL_VLDL_P | Lipoprotein subclasses |  |
| 83 | Total lipids in very large VLDL | XL_VLDL_L | Lipoprotein subclasses |  |
| 84 | Phospholipids in very large VLDL | XL_VLDL_PL | Lipoprotein subclasses |  |
| 85 | Cholesterol in very large VLDL | XL_VLDL_C | Lipoprotein subclasses |  |
| 86 | Cholesteryl esters in very large VLDL | XL_VLDL_CE | Lipoprotein subclasses |  |
| 87 | Free cholesterol in very large VLDL | XL_VLDL_FC | Lipoprotein subclasses |  |
| 88 | Triglycerides in very large VLDL | XL_VLDL_TG | Lipoprotein subclasses |  |
| 89 | Concentration of large VLDL particles | L_VLDL_P | Lipoprotein subclasses |  |
| 90 | Total lipids in large VLDL | L_VLDL_L | Lipoprotein subclasses |  |
| 91 | Phospholipids in large VLDL | L_VLDL_PL | Lipoprotein subclasses |  |
| 92 | Cholesterol in large VLDL | L_VLDL_C | Lipoprotein subclasses |  |
| 93 | Cholesteryl esters in large VLDL | L_VLDL_CE | Lipoprotein subclasses |  |
| 94 | Free cholesterol in large VLDL | L_VLDL_FC | Lipoprotein subclasses |  |
| 95 | Triglycerides in large VLDL | L_VLDL_TG | Lipoprotein subclasses |  |
| 96 | Concentration of medium VLDL particles | M_VLDL_P | Lipoprotein subclasses |  |
| 97 | Total lipids in medium VLDL | M_VLDL_L | Lipoprotein subclasses |  |
| 98 | Phospholipids in medium VLDL | M_VLDL_PL | Lipoprotein subclasses |  |
| 99 | Cholesterol in medium VLDL | M_VLDL_C | Lipoprotein subclasses |  |
| 100 | Cholesteryl esters in medium VLDL | M_VLDL_CE | Lipoprotein subclasses |  |
| 101 | Free cholesterol in medium VLDL | M_VLDL_FC | Lipoprotein subclasses |  |
| 102 | Triglycerides in medium VLDL | M_VLDL_TG | Lipoprotein subclasses |  |
| 103 | Concentration of small VLDL particles | S_VLDL_P | Lipoprotein subclasses |  |
| 104 | Total lipids in small VLDL | S_VLDL_L | Lipoprotein subclasses |  |
| 105 | Phospholipids in small VLDL | S_VLDL_PL | Lipoprotein subclasses |  |
| 106 | Cholesterol in small VLDL | S_VLDL_C | Lipoprotein subclasses |  |
| 107 | Cholesteryl esters in small VLDL | S_VLDL_CE | Lipoprotein subclasses |  |
| 108 | Free cholesterol in small VLDL | S_VLDL_FC | Lipoprotein subclasses |  |
| 109 | Triglycerides in small VLDL | S_VLDL_TG | Lipoprotein subclasses |  |
| 110 | Concentration of very small VLDL particles | XS_VLDL_P | Lipoprotein subclasses |  |
| 111 | Total lipids in very small VLDL | XS_VLDL_L | Lipoprotein subclasses |  |
| 112 | Phospholipids in very small VLDL | XS_VLDL_PL | Lipoprotein subclasses |  |
| 113 | Cholesterol in very small VLDL | XS_VLDL_C | Lipoprotein subclasses |  |
| 114 | Cholesteryl esters in very small VLDL | XS_VLDL_CE | Lipoprotein subclasses |  |
| 115 | Free cholesterol in very small VLDL | XS_VLDL_FC | Lipoprotein subclasses |  |
| 116 | Triglycerides in very small VLDL | XS_VLDL_TG | Lipoprotein subclasses |  |
| 117 | Concentration of IDL particles | IDL_P | Lipoprotein subclasses |  |
| 118 | Total lipids in IDL | IDL_L | Lipoprotein subclasses |  |
| 119 | Phospholipids in IDL | IDL_PL | Lipoprotein subclasses |  |
| 120 | Cholesterol in IDL | IDL_C | Lipoprotein subclasses |  |
| 121 | Cholesteryl esters in IDL | IDL_CE | Lipoprotein subclasses |  |
| 122 | Free cholesterol in IDL | IDL_FC | Lipoprotein subclasses |  |
| 123 | Triglycerides in IDL | IDL_TG | Lipoprotein subclasses |  |
| 124 | Concentration of large LDL particles | L_LDL_P | Lipoprotein subclasses |  |
| 125 | Total lipids in large LDL | L_LDL_L | Lipoprotein subclasses |  |
| 126 | Phospholipids in large LDL | L_LDL_PL | Lipoprotein subclasses |  |
| 127 | Cholesterol in large LDL | L_LDL_C | Lipoprotein subclasses |  |
| 128 | Cholesteryl esters in large LDL | L_LDL_CE | Lipoprotein subclasses |  |
| 129 | Free cholesterol in large LDL | L_LDL_FC | Lipoprotein subclasses |  |
| 130 | Triglycerides in large LDL | L_LDL_TG | Lipoprotein subclasses |  |
| 131 | Concentration of medium LDL particles | M_LDL_P | Lipoprotein subclasses |  |
| 132 | Total lipids in medium LDL | M_LDL_L | Lipoprotein subclasses |  |
| 133 | Phospholipids in medium LDL | M_LDL_PL | Lipoprotein subclasses |  |
| 134 | Cholesterol in medium LDL | M_LDL_C | Lipoprotein subclasses |  |
| 135 | Cholesteryl esters in medium LDL | M_LDL_CE | Lipoprotein subclasses |  |
| 136 | Free cholesterol in medium LDL | M_LDL_FC | Lipoprotein subclasses |  |
| 137 | Triglycerides in medium LDL | M_LDL_TG | Lipoprotein subclasses |  |
| 138 | Concentration of small LDL particles | S_LDL_P | Lipoprotein subclasses |  |
| 139 | Total lipids in small LDL | S_LDL_L | Lipoprotein subclasses |  |
| 140 | Phospholipids in small LDL | S_LDL_PL | Lipoprotein subclasses |  |
| 141 | Cholesterol in small LDL | S_LDL_C | Lipoprotein subclasses |  |
| 142 | Cholesteryl esters in small LDL | S_LDL_CE | Lipoprotein subclasses |  |
| 143 | Free cholesterol in small LDL | S_LDL_FC | Lipoprotein subclasses |  |
| 144 | Triglycerides in small LDL | S_LDL_TG | Lipoprotein subclasses |  |
| 145 | Concentration of very large HDL particles | XL_HDL_P | Lipoprotein subclasses |  |
| 146 | Total lipids in very large HDL | XL_HDL_L | Lipoprotein subclasses |  |
| 147 | Phospholipids in very large HDL | XL_HDL_PL | Lipoprotein subclasses |  |
| 148 | Cholesterol in very large HDL | XL_HDL_C | Lipoprotein subclasses |  |
| 149 | Cholesteryl esters in very large HDL | XL_HDL_CE | Lipoprotein subclasses |  |
| 150 | Free cholesterol in very large HDL | XL_HDL_FC | Lipoprotein subclasses |  |
| 151 | Triglycerides in very large HDL | XL_HDL_TG | Lipoprotein subclasses |  |
| 152 | Concentration of large HDL particles | L_HDL_P | Lipoprotein subclasses |  |
| 153 | Total lipids in large HDL | L_HDL_L | Lipoprotein subclasses |  |
| 154 | Phospholipids in large HDL | L_HDL_PL | Lipoprotein subclasses |  |
| 155 | Cholesterol in large HDL | L_HDL_C | Lipoprotein subclasses |  |
| 156 | Cholesteryl esters in large HDL | L_HDL_CE | Lipoprotein subclasses |  |
| 157 | Free cholesterol in large HDL | L_HDL_FC | Lipoprotein subclasses |  |
| 158 | Triglycerides in large HDL | L_HDL_TG | Lipoprotein subclasses |  |
| 159 | Concentration of medium HDL particles | M_HDL_P | Lipoprotein subclasses |  |
| 160 | Total lipids in medium HDL | M_HDL_L | Lipoprotein subclasses |  |
| 161 | Phospholipids in medium HDL | M_HDL_PL | Lipoprotein subclasses |  |
| 162 | Cholesterol in medium HDL | M_HDL_C | Lipoprotein subclasses |  |
| 163 | Cholesteryl esters in medium HDL | M_HDL_CE | Lipoprotein subclasses |  |
| 164 | Free cholesterol in medium HDL | M_HDL_FC | Lipoprotein subclasses |  |
| 165 | Triglycerides in medium HDL | M_HDL_TG | Lipoprotein subclasses |  |
| 166 | Concentration of small HDL particles | S_HDL_P | Lipoprotein subclasses |  |
| 167 | Total lipids in small HDL | S_HDL_L | Lipoprotein subclasses |  |
| 168 | Phospholipids in small HDL | S_HDL_PL | Lipoprotein subclasses |  |
| 169 | Cholesterol in small HDL | S_HDL_C | Lipoprotein subclasses |  |
| 170 | Cholesteryl esters in small HDL | S_HDL_CE | Lipoprotein subclasses |  |
| 171 | Free cholesterol in small HDL | S_HDL_FC | Lipoprotein subclasses |  |
| 172 | Triglycerides in small HDL | S_HDL_TG | Lipoprotein subclasses |  |

Abbreviations: HDL – high-density lipoprotein; LDL low-density lipoprotein; VLDL – very low-density lipoprotein; IDL – intermediate-density lipoprotein.

**Table S2** List of 51 metabolites identified by urine metabolomics

| **No** | **Urine metabolites** | **Short name** | **Group** | |
| --- | --- | --- | --- | --- |
| 1 | Alanine | ala | Amino acids |  |
| 2 | Glutamine | gln | Amino acids |  |
| 3 | Glycine | gly | Amino acids |  |
| 4 | Isoleucine | ile | Amino acids |  |
| 5 | Leucine | leu | Amino acids |  |
| 6 | Taurine | tau | Amino acids |  |
| 7 | Threonine | thre | Amino acids |  |
| 8 | Tryptophan | trp | Amino acids |  |
| 9 | Tyrosine | tyr | Amino acids |  |
| 10 | Valine | val | Amino acids |  |
| 11 | 2-Hydroxyisobutyrate | aohibut | Amino acid metabolism |  |
| 12 | 3-Hydroxyisobutyrate | bohibut | Amino acid metabolism |  |
| 13 | 3-Hydroxyisovalerate | bohival | Amino acid metabolism |  |
| 14 | 4-Deoxyerythronate | doeta | Amino acid metabolism |  |
| 15 | 4-Deoxythreonate | dta | Amino acid metabolism |  |
| 16 | Ethanolamine | etnh | Amino acid metabolism |  |
| 17 | Glycolate | glya | Amino acid metabolism |  |
| 18 | Hippurate | hip | Amino acid metabolism |  |
| 19 | Pyroglutamate | pglu | Amino acid metabolism |  |
| 20 | Urea | ure | Amino acid metabolism |  |
| 21 | 2-Furoylglycine | furgly | Dietary metabolites |  |
| 22 | Arabinose | arb | Dietary metabolites |  |
| 23 | Ethanol | etoh | Dietary metabolites |  |
| 24 | Mannitol | mnt | Dietary metabolites |  |
| 25 | Proline betaine | probet | Dietary metabolites |  |
| 26 | Propylene glycol | prgly | Dietary metabolites |  |
| 27 | Quinate | quina | Dietary metabolites |  |
| 28 | Sucrose | scr | Dietary metabolites |  |
| 29 | Xylose | xyl | Dietary metabolites |  |
| 30 | Citrate | cit | Energy metabolism |  |
| 31 | Glucose | glc | Energy metabolism |  |
| 32 | Lactate | lac | Energy metabolism |  |
| 33 | cis-Aconitate | caco | Energy metabolism |  |
| 34 | trans-Aconitate | taco | Energy metabolism |  |
| 35 | 3-Hydroxyhippurate | mohhip | Microbial metabolism |  |
| 36 | 4-Hydroxyhippurate | pohhip | Microbial metabolism |  |
| 37 | Acetate | ace | Microbial metabolism |  |
| 38 | Dimethylamine | dma | Microbial metabolism |  |
| 39 | Formate | form | Microbial metabolism |  |
| 40 | HPHPA: 3-(3-Hydroxyphenyl)-3-hydroxypropanate | hphpa | Microbial metabolism |  |
| 41 | Indoxyl Sulfate | ind | Microbial metabolism |  |
| 42 | Trimethylamine N-oxide (TMAO) | tmao | Microbial metabolism |  |
| 43 | 1-Methylnicotinamide | omna | Nicotinate and nicotinamide metabolism |  |
| 44 | Trigonelline | trig | Nicotinate and nicotinamide metabolism |  |
| 45 | 3-Aminoisobutyrate | bnhibut | Nucleotide metabolism |  |
| 46 | Allantoin | aln | Nucleotide metabolism |  |
| 47 | Hypoxanthine | hyp | Nucleotide metabolism |  |
| 48 | Pseudouridine | pseur | Nucleotide metabolism |  |
| 49 | Uracil | ura | Nucleotide metabolism |  |
| 50 | Xanthosine | xan | Nucleotide metabolism |  |
| 51 | Creatinine | crea_abs | Fluid balance |  |

**Table S3** Medication use among study participants

| Drug | Controls (n=28) | m.3243A>G carriers (n=28) |
| --- | --- | --- |
|  |  |  |
| **Blood pressure lowering** |  |  |
| Angiotensin convering enzyme inhibitor/Angiotensin receptor blocker, n (%) | 0 (0%) | 11 (39%) |
| Beta-blockers, n (%) | 0 (0%) | 3 (11%) |
| Calcium channel blockers, n (%) | 0 (0%) | 4 (14%) |
| Diuretics, n (%) | 0 (0%) | 3 (11%) |
| **Glucose lowering** |  |  |
| Dipeptidyl peptidase-4 inhibitors, n (%) | 0 (0%) | 5 (18%) |
| Glucagon-like peptide 1 analogs, n (%) | 0 (0%) | 1 (4%) |
| Insulin, n (%) | 0 (0%) | 9 (32%) |
| Metformin, n (%) | 0 (0%) | 2 (7%) |
| Sodium-glucose transport protein 2 inhibitors, n (%) | 0 (0%) | 1 (4%) |
| Sulfonylurea, n (%) | 0 (0%) | 1 (4%) |
| **Lipid lowering** |  |  |
| Ezetemibe, n (%) | 0 (0%) | 3 (11%) |
| Statins, n (%) | 2 (7%) | 7 |
| **Dietary supplements** |  |  |
| Calcium, n (%) | 2 (7%) | 6 (21%) |
| CoEnzyme Q10, n (%) | 0 (0%) | 1 (4%) |
| Fish oil, n (%) | 4 (14%) | 2 (7%) |
| Magnesium, n (%) | 2 (7%) | 1 (4%) |
| Multivitamin, n (%) | 6 (21%) | 6 (21%) |
| Vitamin B, n (%) | 0 (0%) | 3 (11%) |
| Vitamin D, n (%) | 5 (18%) | 8 (29%) |
| **Others** |  |  |
| Antihistamines, n (%) | 1 (4%) | 2 (7%) |
| Antipsychotics, n (%) | 0 (0%) | 3 (11%) |
| Alpha-blockers, n (%) | 0 (0%) | 1 (4%) |
| Arginine supplement, n (%) | 0 (0%) | 1 (4%) |
| Bisphosphonates, n (%) | 1 (4%) | 3 (11%) |
| Bronchodilators, n (%) | 0 (0%) | 4 (14%) |
| Denosumab, n (%) | 0 (0%) | 1 (4%) |
| Gabapentin, n (%) | 0 (0%) | 1 (4%) |
| Laxatives, n (%) | 0 (0%) | 5 (18%) |
| Lamotrigine, n (%) | 0 (0%) | 1 (4%) |
| Leukotriene receptor antagonists, n (%) | 0 (0%) | 2 (7%) |
| Lithium citrate, n (%) | 0 (0%) | 1 (4%) |
| Melatonin, n (%) | 0 (0%) | 1 (4%) |
| Methotrexate, n (%) | 0 (0%) | 1 (4%) |
| Opioids, n (%) | 0 (0%) | 1 (4%) |
| Nasal corticosteroids, n (%) | 1 (4%) | 4 (14%) |
| Nonsteroidal anti-inflammatories, n (%) | 0 (0%) | 3 (11%) |
| Paracetamol, n (%) | 0 (0%) | 5 (18%) |
| Potassium supplement, n (%) | 0 (0%) | 2 (7%) |
| Proton-pump inhibitors, n (%) | 0 (0%) | 3 (11%) |
| Selective serotonin reuptake inhibitors, n (%) | 0 (0%) | 1 (4%) |
| Serotonin-norepinephrine reuptake inhibitors, n (%) | 0 (0%) | 1 (4%) |
| Skeletal muscle relaxants, n (%) | 0 (0%) | 1 (4%) |
| Tricyclic antidepressants, n (%) | 0 (0%) | 1 (4%) |

**Table S4** Demographic and clinical characteristics of m.3243A>G carriers with and without diabetes.

|  | m.3243A>G carriers without diabetes | m.3243A>G carriers with diabetes | p value |
| --- | --- | --- | --- |
| **Basic characteristics** |  |  |  |
| Number, *n* | 12 | 16 |  |
| Sex (Female/Male) | 6/6 | 12/4 | 0.17 |
| Age (years) | 36.1 ± 12.4 | 47.1 ± 11.2 | **0.02** |
| **Physical examination** |  |  |  |
| Weight (kg) | 65.8 (57.5-79.1) | 56.2 (48.7-72.2) | 0.09 |
| Height (cm) | 171.3 ± 10.4 | 164.9 ± 7.5 | 0.06 |
| Body mass index (kg/m^2^) | 23.3 (20.5-26.6) | 20.5 (18.2-26.2) | 0.29 |
| **Biochemistry** |  |  |  |
| Heteroplasmy in blood (%) | 23.1 ± 14.2 | 24.1 ± 14.0 | 0.86 |
| HbA1c (mmol/mol) | 35.5 (31.5-40.5) | 50.0 (44.5-58.5) | **<0.001** |
| Cholesterol (mmol/L) | 4.6 ± 0.8 | 4.3 ± 0.8 | 0.22 |
| HDL (mmol/L) | 1.2 ± 0.4 | 1.2 ± 0.3 | 0.94 |
| LDL (mmol/L) | 2.7 ± 0.7 | 2.4 ± 0.7 | 0.23 |
| Triglycerides (mmol/L) | 1.5 (1.4-1.8) | 1.3 (1.0-1.7) | 0.54 |
| Creatinine (µmol/L) | 66.7 ± 10.8 | 74.1 ±18.0 | 0.22 |
| ALAT (U/L) | 37.0 (24.5-52.0) | 24.5 (18.0-35.5) | 0.09 |
| ASAT (U/L) | 26.0 (24.0-31.0) | 24.5 (19.0-28.0) | 0.11 |
| Alkaline phosphatase (U/L) | 70.6 ± 16.9 | 82.8 ± 24.9 | 0.16 |
| **Diabetes medication^a^** |  |  |  |
| Insulin (yes), n (%) | - | 9 (56%) | - |
| Oral antidiabetics (yes), n (%) | - | 6 (38%) | - |
| Dipeptidyl peptidase-4 inhibitors (yes), n (%) | - | 5 (31%) | - |
| Metformin (yes), n (%) | - | 2 (13%) | - |
| Sulfonylurea (yes), n (%) | - | 1 (6%) | - |
| SGLT2 | - | 1 (6%) | - |
| **Symptomatic assessment** |  |  |  |
| Duration of diabetes (years) | - | 13.7 ± 8.5 | - |
| Ataxia, n (%) | 0 (0%) | 3 (19%) | 0.11 |
| Epilepsy, n (%) | 0 (0%) | 0 (0%) | - |
| Stroke-like episodes, n (%) | 0 (0%) | 0 (0%) | - |
| Peripheral neuropathy, n (%) | 4 (33%) | 8 (50%) | 0.38 |
| Myopathy, n (%) | 5 (42%) | 13 (81%) | **0.03** |
| Hearing impairment, n (%) | 5 (42%) | 14 (88%) | **0.01** |
| Cardiomyopathy, n (%) | 0 (0%) | 5 (31%) | **0.03** |
| Hypertension, n (%) | 2 (17%) | 8 (50%) | 0.07 |
| Non-diabetic nephropathy, n (%) | 1 (8%) | 1 (6%) | 0.83 |

Statistically significant differences are marked in bold.

^a^For full list of medication see Table S3.

Abbreviations: HbA1c – haemoglobin A1C; HDL – high-density lipoprotein; LDL – low-density lipoprotein; ALAT – alanine aminotransferase; ASAT – aspartate aminotransferase; SGLT2 - Sodium Glucose Cotransporter 2.

**Table S5** Twelve metabolites that are significantly different between at least one pair of groups revealed by the one-way analysis of variance (ANOVA) with Fisher’s least significant difference test in urine metabolomics

| **Metabolites** | **f value** | **Unadjusted p value (ANOVA)** | **FDR^a^** | **p value**  **(CTRL-Non-DIAB)** | **p value**  **(CTRL-DIAB)** | **p value**  **(NON-DIAB – DIAB)** |
| --- | --- | --- | --- | --- | --- | --- |
| Uracil | 18.846 | < 0.001 | < 0.001 | <0.05 | <0.05 | NS |
| 1-Methylnicotinamide | 16.083 | < 0.001 | < 0.001 | <0.05 | <0.05 | <0.05 |
| Hypoxanthine | 12.807 | < 0.001 | < 0.001 | <0.05 | <0.05 | NS |
| Pseudouridine | 10.902 | < 0.001 | 0.001 | NS | <0.05 | <0.05 |
| Creatinine | 10.814 | < 0.001 | 0.001 | NS | <0.05 | <0.05 |
| Ethanolamine | 8.383 | < 0.001 | 0.006 | NS | <0.05 | <0.05 |
| Dimethylamine | 8.233 | < 0.001 | 0.006 | NS | <0.05 | NS |
| Glycine | 7.619 | 0.001 | 0.008 | <0.05 | <0.05 | NS |
| 4-Deoxyerythronate | 6.904 | 0.002 | 0.012 | NS | <0.05 | <0.05 |
| Arabinose | 6.830 | 0.002 | 0.012 | NS | <0.05 | <0.05 |
| Glucose | 5.219 | 0.009 | 0.039 | NS | <0.05 | <0.05 |
| Pyroglutamate | 5.138 | 0.009 | 0.039 | NS | <0.05 | NS |

^a^False discovery rate (FDR) represents the p-value after adjusting for multiple comparisons.

Abbreviations: CTRL – controls; DIAB – m.3243A>G carriers with diabetes; Non-DIAB – m.3243A>G carriers without diabetes; FDR – false discovery rate; NS – non-significant.

**Table S6** Prediction models distinguishing between m.3243A>G carriers with and without diabetes (A) and healthy controls and m.3243A>G carriers without diabetes (B)

| **A: Prediction models based on a set of three urine metabolites with the highest AUC** | | | |
| --- | --- | --- | --- |
| **Machine learning algorithm** | **AUC** | **p value** | **Cross-validation Prediction** |
| Linear support vector | 0.84 (0.55-1) | 0.035 | 0.713 |
| PLS-DA | 0.81 (0.59-1) | 0.045 | 0.711 |
| Logistic regression | 0.80 (0.52-1) | 0.050 | 0.722 |
| Random forest | 0.76 (0.56-0.95) | 0.077 | 0.679 |

| **B: Prediction models based on a set of four urine metabolites with the highest AUC** | | | |
| --- | --- | --- | --- |
| **Algorithm** | **AUC** | **p value** | **Cross-validation Prediction** |
| Linear support vector | 0.95 (0.81-1) | < 0.001 | 0.82 |
| PLS-DA | 0.90 (0.74-1) | 0.001 | 0.78 |
| Logistic regression | 0.94 (0.78-1) | 0.001 | 0.90 |
| Random forest | 0.86 (0.63-0.99) | 0.005 | 0.79 |

Abbreviations: AUC – area under the curve; PLS-DA – Partial Least Squares Discriminant Analysis.

**Table S7** Set of serum and urine metabolites to distinguish m.3243A>G carriers with diabetes from healthy controls.

| **Serum metabolites^a^** | | **AUC** | | **p value** | **Log2 FC** |  |
| --- | --- | --- | --- | --- | --- | --- |
| Triglycerides in LDL | | 0.82 | | 0.002 | -0.37 |  |
| Triglycerides in medium LDL | | 0.82 | | 0.002 | -0.42 |  |
| Triglycerides in very small VLDL | | 0.81 | | 0.002 | -0.45 |  |
| Triglycerides in HDL | | 0.81 | | <0.001 | -0.50 |  |
| Triglycerides in IDL | | 0.81 | | 0.003 | -0.34 |  |
| Triglycerides in large LDL | | 0.80 | | 0.003 | -0.33 |  |
| Pyruvate | | 0.79 | | <0.001 | -0.59 |  |
| Triglycerides in medium HDL | | 0.78 | | 0.001 | -0.52 |  |
| Triglycerides in small LDL | | 0.78 | | 0.003 | -0.54 |  |
| Triglycerides in very large HDL | | 0.77 | | 0.002 | -0.55 |  |
| Glucose | | 0.77 | | 0.019 | -0.38 |  |
| Monounsaturated fatty acids | | 0.77 | | 0.003 | -0.38 |  |
| Concentration of VLDL particles | | 0.77 | | 0.008 | -0.38 |  |
| Polyunsaturated fatty acids/Monounsaturated fatty acids | | 0.77 | | 0.002 | 0.23 |  |
| Triglycerides in small HDL | | 0.76 | | 0.002 | -0.49 |  |
| Total lipids in small VLDL | | 0.76 | | 0.012 | -0.38 |  |
| Lactate | | 0.76 | | 0.001 | -0.54 |  |
| Concentration of small VLDL particles | | 0.76 | | 0.012 | -0.40 |  |
| Degree of unsaturation | | 0.76 | | 0.002 | 0.07 |  |
| Linoleic acid | | 0.75 | | 0.026 | -0.11 |  |
| Concentration of very small VLDL particles | | 0.75 | | 0.028 | -0.22 |  |
| Phospholipids in very small VLDL | | 0.74 | | 0.023 | -0.27 |  |
| Triglycerides in large HDL | | 0.74 | | 0.018 | -0.46 |  |
| Phospholipids in VLDL | | 0.74 | | 0.009 | -0.52 |  |
| Phospholipids in small VLDL | | 0.73 | | 0.028 | -0.30 |  |
| Cholesteryl esters in small VLDL | | 0.73 | | 0.047 | -0.29 |  |
| Saturated fatty acids | | 0.73 | | 0.004 | -0.31 |  |
| Total lipids in very small VLDL | | 0.73 | | 0.025 | -0.24 |  |
| Total fatty acids | | 0.73 | | 0.005 | -0.24 |  |
| Cholesterol in VLDL | | 0.73 | | 0.017 | -0.36 |  |
| Free cholesterol in VLDL | | 0.73 | | 0.013 | -0.46 |  |
| Cholesteryl esters in VLDL | | 0.72 | | 0.030 | -0.29 |  |
| Triglycerides in small VLDL | | 0.72 | | 0.007 | -0.54 |  |
| Total triglycerides | | 0.72 | | 0.008 | -0.65 |  |
| Total lipids in VLDL | | 0.72 | | 0.011 | -0.58 |  |
| Cholesteryl esters in very large VLDL | | 0.71 | | 0.017 | -0.60 |  |
| Cholesterol in small VLDL | | 0.71 | | 0.057 | -0.26 |  |
| Cholesteryl esters in large VLDL | | 0.71 | | 0.019 | -0.60 |  |
| Free cholesterol in very small VLDL | | 0.71 | | 0.051 | -0.21 |  |
| Total lipids in medium VLDL | | 0.71 | | 0.023 | -0.41 |  |
| Omega-6 fatty acids | | 0.70 | | 0.078 | -0.08 |  |
| Phospholipids in chylomicrons and extremely large VLDL | | 0.70 | | 0.025 | -1.67 |  |
| Cholesteryl esters in chylomicrons and extremely large VLDL | | 0.70 | | 0.012 | -1.18 |  |
| Phospholipids in medium VLDL | | 0.70 | | 0.028 | -0.37 |  |
| **Urine metabolites** | | **AUC** | | **p value** | **Log2 FC** |  |
| Uracil | | 0.96 | | < 0.001 | 1.89 |  |
| Hypoxanthine | | 0.95 | | < 0.001 | 3.12 |  |
| 1-Methylnicotinamide | | 0.92 | | < 0.001 | 1.54 |  |
| Creatinine | | 0.88 | | < 0.001 | 1.41 |  |
| Pseudouridine | | 0.87 | | < 0.001 | 1.34 |  |
| Ethanolamine | | 0.84 | | < 0.001 | 1.43 |  |
| Dimethylamine | | 0.83 | | < 0.001 | 1.05 |  |
| Deoxyerythronate | | 0.82 | | < 0.001 | 1.07 |  |
| Glycine | | 0.82 | | 0.001 | 1.47 |  |
| Proline betaine | | 0.82 | | 0.009 | 2.16 |  |
| 2-Hydroxyisobutyrate | | 0.80 | | 0.006 | 0.98 |  |
| Threonine | | 0.78 | | 0.006 | 0.94 |  |
| Pyroglutamate | | 0.78 | | 0.002 | 0.93 |  |
| 4-Hydroxyhippurate | | 0.78 | | 0.014 | 1.21 |  |
| Deoxythreonate | | 0.77 | | 0.008 | 1.05 |  |
| Tryptophan | | 0.74 | | 0.018 | 0.81 |  |
| Glycolate | | 0.72 | | 0.042 | 0.73 |  |
| Arabinose | | 0.71 | | 0.004 | -1.75 |  |
| **Prediction models based on a set of 3 urine metabolites with highest AUC and valid p value** | | | | | | |
| **Algorithm** | | **AUC** | | **P value** | | **Cross-validation Prediction** |
| Linear support vector | | 0.98 (0.93-1) | | < 0.001 | | 0.855 |
| PLS-DA | | 0.97 (0.90-1) | | < 0.001 | | 0.814 |
| Logistic regression | | 0.94 (0.71-1) | | 0.001 | | 0.893 |
| Random forest | | 0.99 (0.92-1) | | < 0.001 | | 0.928 |

^a^Using 24 serum metabolites PLS-DA could discriminate between groups with AUC=0.743 and p=0.038, while linear support vector, logistic regression and random forest machine learning algorithms were not able to discriminate between groups (AUC<0.7).

Abbreviations: AUC- area under the curve; FC – fold change; HDL – high-density lipoprotein; LDL low-density lipoprotein; VLDL – very low-density lipoprotein; IDL – intermediate-density lipoprotein; PLS-DA – Partial Least Squares Discriminant Analysis.
